# Measuring Seclusion in Psychiatric Intensive Care: Development and Measurement Properties of the Clinical Seclusion Checklist

**DOI:** 10.3389/fpsyt.2021.768500

**Published:** 2021-12-23

**Authors:** Torleif Ruud, Espen Woldsengen Haugom, Harold Alan Pincus, Torfinn Hynnekleiv

**Affiliations:** ^1^Mental Health Services, Akershus University Hospital, Lørenskog, Norway; ^2^Institute of Clinical Medicine, University of Oslo, Oslo, Norway; ^3^Department of Acute Psychiatry and Psychosis Treatment, Sanderud, Division of Mental Health, Innlandet Hospital Trust, Ottestad, Norway; ^4^Department of Psychiatry and Irving Institute for Clinical and Translational Research, Columbia University, New York City, NY, United States; ^5^New York State Psychiatric Institute, New York City, NY, United States; ^6^Department of Acute Psychiatry and Psychosis Treatment, Division of Mental Health, Innlandet Hospital Trust, Reinsvoll, Norway

**Keywords:** clinical seclusion checklist, seclusion, psychiatric intensive care, psychiatric acute wards, emergency psychiatry, checklist, measuring, measurement properties

## Abstract

**Background:** Acute psychiatric units in general hospitals must ensure that acutely disturbed patients do not harm themselves or others, and simultaneously provide care and treatment and help patients regain control of their behavior. This led to the development of strategies for the seclusion of a patient in this state within a particular area separated from other patients in the ward. While versions of this practice have been used in different countries and settings, a systematic framework for describing the various parameters and types of seclusion interventions has not been available. The aims of the project were to develop and test a valid and reliable checklist for characterizing seclusion in inpatient psychiatric care.

**Methods:** Development and testing of the checklist were accomplished in five stages. Staff in psychiatric units completed detailed descriptions of seclusion episodes. Elements of seclusion were identified by thematic analysis of this material, and consensus regarding these elements was achieved through a Delphi process comprising two rounds. Good content validity was ensured through the sample of seclusion episodes and the representative participants in the Delphi process. The first draft of the checklist was revised based on testing by clinicians assessing seclusion episodes. The revised checklist with six reasons for and 10 elements of seclusion was tested with different response scales, and acceptable interrater reliability was achieved.

**Results:** The Clinical Seclusion Checklist is a brief and feasible tool measuring six reasons for seclusion, 10 elements of seclusion, and four contextual factors. It was developed through a transparent process and exhibited good content validity and acceptable interrater reliability.

**Conclusion:** The checklist is a step toward achieving valid and clinically relevant measurements of seclusion. Its use in psychiatric units may contribute to quality assurance, more reliable statistics and comparisons across sites and periods, improved research on patients' experiences of seclusion and its effects, reduction of negative consequences of seclusion, and improvement of psychiatric intensive care.

## Introduction

Acute psychiatric units in general hospitals must give emergency care to people with various psychiatric conditions, including acutely disturbed patients representing a risk of harming themselves, other patients, or clinical staff (1, 2). A major challenge has been the conflicting tasks of controlling behavior and securing safety for these patients and others, while simultaneously providing a therapeutic milieu and intensive treatment for mental illness.

To meet this challenge, acute psychiatric units have developed models of care that combine keeping the most disturbed patients separated from other patients and, at the same time, providing intensive psychiatric care and treatment. The term “seclusion” is used in the literature to denote keeping patients separated from other patients and usually in a locked room without staff present, and seclusion is a part of different models of psychiatric intensive care that have been developed.

### Intensive Care Models With Seclusion

The psychiatric intensive care unit (PICU) is the most well-known model (1, 3). The PICU is usually a small unit with a few beds and a high staff-to-patient ratio. The unit aims to meet patients' needs for personal space within a safe and secure setting with limited stimuli. Care is provided by a multidisciplinary team with a high level of competence in teamwork, violence prevention, “talking down” acutely disturbed patients, respecting patients, supporting patient autonomy, providing daily structure and other elements of milieu therapy. Treatment often also includes psychotropic medication. Psychodynamic and/or cognitive-behavioral training and supervision are often given to help the staff understand these patients, their reactions to the patients, and what these reactions tells about the patients' problems and needs. Reviews have identified variations in the PICU practice, as well as a lack of empirical data about its practice and outcomes (1, 3). The implementation of some elements of the PICU model has been reported in one review (4).

The High and Intensive Care (HIC) model was developed in the Netherlands over the past decade, building partly on the PICU model (5). This model is based on a stepped-care approach within a psychiatric ward: Patients are admitted to a high care unit (HC) and, further, to an intensive care unit (IC) for a maximum of three days if needed due to aggression. The IC does not have its own staff, so the HC staff follow these patients while they are in the IC. The IC also has a high-security room that is locked and is a coercive measure. The HIC Monitor fidelity scale has been developed and tested (6), and implementation of the HIC model has recently been reported (7).

Safewards is a model designed to reduce violence and the use of containment (8–10). The model consists of 10 interventions designed to address documented causes of violence and of use of containment. These interventions are specific staff interactions tailored to different types of situations with patients. Safewards is related to the PICU and HIC models, and the Safewards interventions may be integrated with these and other psychiatric intensive care models.

In Norway, facing the same challenges as described above, the mental health services also developed a version of seclusion (*skjerming*, a Norwegian word meaning protection or shielding) in psychiatric intensive care as an extension of milieu therapy (2). According to the Norwegian Mental Health Care Act, *skjerming* is keeping the patient separated from other patients but with staff present (11), much like isolation in a locked area, accompanied by nurses, which sometimes has been called “open area seclusion” (12, 13). This is in contrast to the isolation of a patient in a locked room without staff, which is highly restricted in Norway and may be used only under exceptional circumstances and, then, limited to a maximum of two hours. The version of seclusion used in Norway was developed as a therapeutic model building on a psychodynamic definition of milieu therapy with containment, support, structure, involvement, and validation as key concepts (14). This model can be applied in psychiatric intensive care, and this has been well-described in one of the reviews cited above (3). Preventing acutely disturbed patients from harming themselves or others while, at the same time, providing more-intensive contact and an individually tailored milieu therapy can include a range of activities in addition to the reduction of stress and sensory stimuli. In Norway, elements of seclusion have also been used in informal voluntary agreements with patients, e.g. when a patient agreed to seclude himself in his room for some hours to avoid stimuli. However, seclusion has, increasingly, been seen as an involuntary coercive measure with a legally formalized decision by a senior clinician and strictly regulated by the Mental Health Care Act (11). Seclusion may be implemented in the patient's room in the ward or a designated seclusion area with a few individual patient rooms. Such areas do not have their own staff, so a patient in seclusion is followed by ward staff that the patient already knows. Seclusion means more access to staff and more intensive care, demanding more resources. However, the law and national guidelines do not describe the content of what the patient and staff do together, which may have led to different ways of practicing seclusion.

### International Variations of Seclusion

There is no established international definition of seclusion. The World Health Organzation (WHO) has recommended that seclusion be defined in national legislation, as there can be various interpretations (15). However, the lack of international consensus makes reliable comparisons difficult, across countries and, often, within them. Recently, a definition of seclusion has been developed in secure residential youth care in the Netherlands through an extensive process involving both health professionals and youth (16). This defines seclusion as “an involuntary placement in a room or area the client is not allowed or able to leave.” This definition of seclusion may also be appropriate for adult mental health services across countries. A strength of the definition is that it is broad, as some of the more-specific details that differ among various definitions did not achieve consensus in the process (16). We consider the Dutch definition useful. It is broad enough to encompass many of the variations of seclusion described in the literature, and is sufficiently operationalizable to be a candidate for international consensus as a definition of seclusion. However, this means that several more detailed aspects of seclusion need to be measured to enable reliable comparisons (16).

The use of seclusion varies across countries and within countries, and reliable comparisons are difficult due to these variations in how seclusion is defined and practiced, and how it is measured and reported (17–20). A review of several larger studies identified up to 110 seclusions per 1,000 inpatient days in the United States and up to 116 seclusions per 1,000 admissions in Europe (17); these figures indicate that a substantial proportion of inpatients in psychiatric units may experience seclusion.

International reviews of seclusion indicated variations in several aspects of how seclusion is practiced or found that studies did not report characteristics of the wards or the seclusion practice (21–23). A systematic review of seclusion in Norway also suggested that there may be differences in how seclusion is understood and practiced (2). Heterogeneity of seclusion practice has been seen for aspects such as the physical environment for seclusion, the presence of staff with the patient, and the duration of seclusion episodes. These aspects were removed from the Dutch definition cited above due to a lack of consensus for these in the last stage of its development (16). A study in the impact of the physical environment of 200 psychiatric wards found that some ward features (presence of outdoor space, special safety measures, large number of patients in the building) increased the risk of being secluded, while some other features (total private space per patient, level of comfort, greater visibility on the ward) decreased the risk of being secluded (24). It is likely that such factors also may have similar effects on patient behaviors during seclusion. One study has found that threatening behavior and violent incidents were lower among patients in seclusion in a PICU than among patients in the acute psychiatric ward (25). While seclusion rooms often have very limited furniture like a bed and a mattress, another study found no significant differences in symptoms or dangerous behavior in a seclusion area with a sparsely interior compared to a seclusion area looking like an ordinary home (26). There are also variations whether doors between seclusion areas and the rest of the ward are locked or open (16). While patients mostly are secluded alone and without staff present, there also seems to be seclusion practices where staff are present with patients all the time or part of the time (23).

Seclusion has been studied and discussed often as a form of coercion, along with physical restraints (21). A recent systematic review of 35 studies on the effects of seclusion and restraints found that both have deleterious physical and psychological effects on patients, and coercion should be used only as a last resort (21). The review was unable to reach a conclusion about beneficial effects of seclusion and restraints, but found that seclusion seemed to be better accepted by patients than other coercive measures and may be perceived as less invasive. The review also indicated that “therapeutic interaction seems to influence perceptions of coercion and could help to avoid negative effects when coercive measures are not avoidable” (21). Another review could not conclude which was superior, seclusion or physical restraint, but did find that patients generally preferred seclusion over physical restraints, while physical restraints seemed to be a safer option for patients exhibiting severe self-harm (22). A review of staff and patient views of seclusion practices found that the majority of staff believed that seclusion was largely beneficial for patients because the patient could calm down and regain control (23). Both staff and patients emphasized the need for more contact and better communication between patient and staff, including explaining procedures and debriefing sessions after the seclusion. The patients wanted the staff to stay with them and provide support during the seclusion. They also wanted the seclusion room to be comfortable and decorated, and to have things they could do while secluded.

Seclusion and other forms of coercion should be avoided in mental health services and only be a last resort, as included in the United Nations Declarations of Human Rights and in standards from the Council of Europe's European Committee for the Prevention of Torture and Inhuman or Degrading Treatment or Punishment (27, 28). A review found that few studies had been done on the complex ethical dilemmas for the staff when seclusion is considered necessary for promoting the patient's best interest (29). This article does not focus on ethical dilemmas, although they are always present when patients are secluded (30). These challenges for the staff are presented and discussed in a separate article published from the current study, based on perspectives of the clinical staff regarding ethical aspects included in their detailed descriptions of the seclusion episodes during stage 1 of the project (see methods) (31). The main finding was that the balance between the staff's sincere desire to provide good treatment and the necessity to control the patient's behavior could be ethically challenging and burdensome, and that working under such conditions may result in psychosocial strain on the staff.

### Mesurement of Seclusion

Most of the studies on seclusion provide little information about the characteristics of the wards or the physical arrangements for seclusion (22). Research on the content of seclusion is even more limited, and the lack of measurement tools is one barrier to advancing such research and knowledge (32). Moreover, few attempts have been made to measure the content of milieu therapy or inpatient psychiatric treatment (33). There is a fidelity scale (the HIC Monitor) measuring the implementation of the HIC model at the ward level (6) and a questionnaire (the Patient-staff Conflict Checklist) designed for use by the head of the unit to measure the use of the Safewards interventions at the ward level (10, 34). We have also found a Self-Assessment Seclusion Checklist that clinical units can use to rate aspects of their own seclusion practice (35). However, we have not found any tool measuring the elements of the seclusion provided to the individual patient.

Thus, there is a clear need to measure various aspects of seclusion, and it is essential to develop a uniform registration system to monitor seclusion and its different dimensions (16). Without a valid and reliable tool for measuring seclusion at the patient level, we cannot determine how it is provided to individual patients or study how the elements and varations of seclusion are related to clinical outcomes and patient experiences. As a result, we may overlook actual differences in seclusion and report differences that are not real, raising a reasonable doubt about whether data on seclusion from different inpatient units could be reliably compared. Measuring different aspects of seclusion and its effects may contribute to reducing its use and its coercive and harmful elements while improving the supportive elements of mental health care and relationships that patients experience as positive and helpful.

### Aims

The project aimed to develop a valid and reliable checklist that can be used to measure seclusion, delineate seclusion elements, compare seclusion practices, and study the effects and experiences of seclusion. To create such a checklist, we needed to operationalize the elements of real world seclusion in terms of measurable variables identifying what is done in seclusion episodes.

## Materials and Methods

### Design and Context

**Figure 1:**
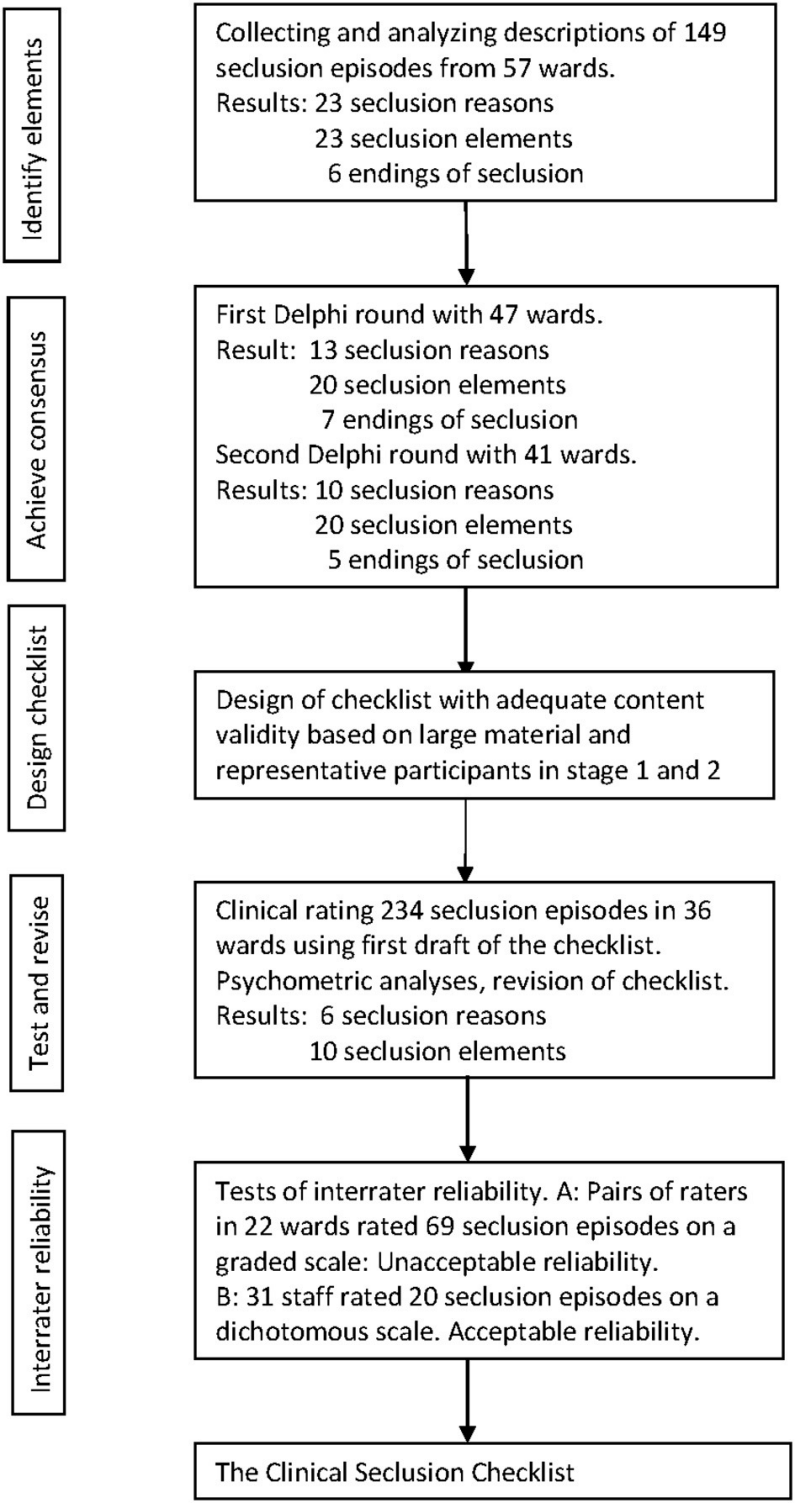
Stages in development and testing of the Clinical Seclusion Checklist.

The development and testing of the checklist were conducted in five stages: identifying elements of seclusion; achieving consensus on elements of seclusion; designing a checklist with good content validity; revising the checklist based on testing in clinical practice; and achieving sufficient interrater reliability of the checklist. An overview of the five stages is shown in Figure 1.

The project was undertaken in 2012–2018 as a project within the national Network for Acute Mental Health Services, where managers and staff from a majority of acute psychiatric units in Norway met twice a year. The project had an advisory group with two persons from users' and relatives' organizations and seven clinical staff members from acute psychiatric units in health trusts in different parts of Norway. The group met at the end of the first and second stages to discuss the results of each stage and the elements that should be included in the next stage.

The project was approved by the Akershus University Hospital Data Protection Officer (reg. no. 2012/095). The Regional Committee for Medical and Health Research Ethics (REK) determined that the project did not need approval from REK because it was a quality project using anonymized data (REK, reg. no. 2013/243). The study followed the protocols for the Declaration of Helsinki.

### Stage 1. Identification of Elements of Seclusion

In 2012, all psychiatric departments in Norway with inpatient wards (units) using seclusion were invited to participate in the project, and 65 wards accepted. Most were acute psychiatric wards, and some were security wards, psychosis wards, wards for adolescents, and wards for older patients.

Each participating ward was asked to provide descriptions of three or more seclusion episodes, and a form was developed for this purpose. The descriptions were provided by the healthcare professionals who were in charge of deciding on seclusion and implementing it, and the completed forms were submitted to the project through a secure online portal. The form was a Word file with sections to describe background and rationale, aims, elements, duration, ways of ending, and ethical aspects of the seclusion episode. Staff on participating wards had provided feedback to a draft of the form, and the project was in dialogue with local project coordinators during the process to provide support and facilitation for the descriptions to be as specific and detailed as possible. An English translation of the form for describing a seclusion episode is included as Supplementary Material (Data Sheet 1).

The first step in the analysis of the descriptions was reading them thoroughly several times to become familiar with the material and gain a sense of wholeness (36). Thematic analysis was then performed by dividing statements into groups by content and developing preliminary codes based on an increasing number of descriptions (37). These codes were grouped into categories of seclusion elements at a higher abstraction level, resulting in a code sheet that was used in the analysis. New codes and categories identified during the remaining analysis were added to the code sheet. The aim was to identify a manageable number of seclusion elements with a suitable abstraction level as separate concepts specific enough without being too detailed. Codes that were variants of the same element (e.g., different ways of reducing stimuli) were pooled and assigned a category name that covered all the variants. A reliability check performed by two project members independently coding 30 randomly selected descriptions using the identified categories indicated acceptable agreement between the two researchers.

### Stage 2. Achieving Consensus on Elements of Seclusion

We used a Delphi process in 2013 to achieve consensus on which identified categories were elements of seclusion and should be included in the checklist (38, 39). Multidisciplinary groups in 47 wards participated in the first round. For each item identified from the analysis of the descriptions of seclusion episodes, the staff of the wards voted on a scale of 1–9 whether they considered the item to be an element of seclusion. They were advised to first choose between low (range 1–3), medium (range 4–6), and high (range 7–9) certainty that the items could be part of seclusion, and then finalize a rating within the chosen range. They were also invited to suggest rephrasing items or propose new items.

In the second round, with 41 wards participating, staff were given information about the distribution of ratings for each item in the first round, including for items that had been excluded due to a high degree of consensus that they could not be considered an element of seclusion. Based on this information, they were invited to vote again on each item, as well as on new items that had been proposed in the first round. For some items, they could also vote for alternative phrasings that had been suggested. A third round could be implemented if necessary to achieve consensus.

### Stage 3. Designing a Checklist With Adequate Content Validity

The seclusion reasons and elements that reached consensus through the Delphi process were defined as items for the first draft of a checklist in 2014. We chose a three-step response scale for reasons for seclusion and for a way of ending seclusion (0 = no reason, 1 = additional reason, 2 = main reason), and a three-step response scale for elements of seclusion (0 = not done, 1 = done some of the time or partially, 2 = done most of the time). The first two stages were expected to provide acceptable content validity for items in the checklist according to a definition of content validity in guidelines for scale developments: “Content validity concerns item sampling adequacy – that is, the extent to which a set of items reflects a content domain” (40).

### Stage 4. Revising the Checklist Based on Testing in Clinical Practice

We tested the clinical relevance of the items of the first draft of the checklist in 2014–2015, giving further support to its content validity. This included measuring how often each element was part of a seclusion episode and whether psychometric analyses of the results could provide a basis for shortening or simplifying the checklist by removing items, merging items, or reformulating items (40). A total of 36 wards participated and rated 234 seclusion episodes.

Revising the checklist, we first used descriptive statistics to identify items that were seldom used. We then conducted exploratory factor analyses and correlation analyses to identify items that measured the same dimension and were so similar that they could be merged. We used principal component analyses with Kaiser's criterion of eigenvalue 1 or above and varimax rotation (40, 41). Internal consistency for factors was analyzed by calculating Cronbach's alpha (42). Finally, we revised the items on the checklist by reformulating or removing items that did not function well and merging items that were quite similar, making the checklist clearer and shorter.

### Stage 5. Testing and Achieving Adequate Interrater Reliability of the Checklist

The fifth and final stage was to test and achieve acceptable interrater reliability for rating the final seclusion reasons and elements. The testing was conducted in 2015–2016 with a dichotomous response scale (yes/no) for reasons and a graded five-step response scale for elements rating how much of the time the element was used in a seclusion episode (from not used to use all the time). A total of 69 seclusion episodes in 22 wards were rated by two clinicians/staff familiar with the specific episode. They rated at the end of the seclusion episode, and they performed the rating independently. As we did not achieve acceptable interrater reliability for the seclusion elements, we adjusted the response scales for these to a dichotomous response scale (yes/no). In 2018–2019 we achieved an acceptable level of interrater reliability with dichotomous response scales, based on 31 clinicians independently rating 20 of the seclusion episode descriptions from stage 1 in the project.

## Results

### Stage 1. Identifying Elements of Seclusion

In all, staff from 57 wards provided systematic and detailed descriptions of a total of 149 seclusion episodes. The descriptions ranged from a half-page to seven and a half pages (average length two and a third pages), and the total material comprised 345 pages.

The thematic analyses of the material identified 23 reasons for seclusion, 23 seclusion elements, and six ways of ending seclusion. These are shown in Supplementary Tables 1A–E from the first Delphi round, as they were the input to the Delphi process. The descriptions of ethical dilemmas experienced while implementing the seclusion episodes are not analyzed in this article as these have been analyzed and published in another article (31).

### Stage 2. Achieving Consensus on Elements of Seclusion

The results of the ratings and conclusions of the first Delphi round are shown in Supplementary Tables 1A–E. The first Delphi round with 47 participating wards resulted in 13 reasons for seclusion, 20 elements of seclusion, and seven ways of ending seclusion. Six new items were proposed. It was not considered necessary to include in the second round four contextual items on seclusion (Supplementary Table 1C) and 22 items on whether the elements of seclusion also could be used in mileu therapy outside seclusion Supplementary Table 1E. There was consensus in the first round that activities and structure and treatment were elements that could be used in milieu therapy outside seclusion, while there was only partly consensus that restrictive elements could be used in milieu therapy outside seclusion. The results of the ratings and conclusions of the second Delphi round are shown in Supplementary Tables 2A–C. The second round with 41 participating wards resulted in consensus on 10 reasons for seclusion, 20 seclusion elements, and five seclusion endings. The second round showed that there was mostly a clear consensus on the elements retained after the first round, and we concluded that there was no need for a third round. We considered the two Delphi rounds as an effective and successful process that achieved a clear consensus on which elements to include in the checklist.

### Stage 3. Designing a Checklist With Adequate Content Validity

Using the 10 seclusion reasons, 20 seclusion elements and five seclusion endings from the Delphi process, we constructed a first draft of the checklist. According to the definition of content delivery quoted above under methods, we considered that adequate content validity of the items in the checklist had been achieved through the large representative sample of seclusion episodes described in detail and analyzed in stage 1 and the large representative sample of ward staff in the Delphi process achieving consensus in stage 2 (40).

### Stage 4. Revising the Checklist Based on Testing in Clinical Practice

The testing of the checklist in clinical practice was conducted by rating 234 seclusion episodes in 36 wards. Table 1 shows the frequency for each of the 35 items in these 234 seclusion episodes. As described in the methods section, we revised the checklist based on factor analysis on each of the three groups of items, identifying factors with similar items that could be merged to replace a group of items and thus reducing the number of items in the checklist. Table 2 identifies the decisions on each of the 35 items based on the statistical analyses and a review of all available information. We decided to remove the section on how the seclusion was ended, as the results of the Delphi process revealed that seclusions were generally discontinued by letting the patient gradually increase time spent outside seclusion without the introduction of any new elements. The revision resulted in a shorter checklist with six seclusion reasons and 10 seclusion elements. These 16 items are displayed in Table 3 and in the final checklist in the Supplementary Material.

**Table 1:** Results from testing the first draft of the checklist rating seclusion episodes (*N* = 234).

	**Reasons for seclusion**	**No reason**	**Additional reason**	**Main reason**
1	The patient's behavior affects other patients in a negative way	46	97	86
2	The patient shows uncritical behavior	30	81	115
3	The patient is intoxicated, and this affects the behavior	184	13	23
4	The patient is violent toward the staff	145	36	40
5	The patient is threatening the staff	96	64	65
6	The patient is violent toward other patients	203	13	3
7	The patient is threatening other patients	183	28	11
8	The patient's behavior is chaotic	30	73	120
9	The patient has significantly increased activity	83	72	68
10	Staff consider that there is a high risk of suicide	196	10	11
	**Elements of seclusion used**	**Not used**	**Part of the time/partly**	**Most of** **the time**
1	Activities with staff during seclusion	57	108	64
2	Activities with staff outside the ward	100	99	30
3	Activities alone during seclusion	99	100	30
4	Support conversations with the patient	14	97	119
5	Reduction of stimuli or sensory impressions	24	59	147
6	Locking of personal belongings	79	78	73
7	Regulation of access to TV, radio, or internet	76	72	83
8	Regulation of contact with relatives	175	38	16
9	Regulation of contact with other patients	33	86	111
10	Regulation of access to mobile phone	159	34	35
11	Restrict access to objects that the patient can use to harm themselves or others	93	54	84
12	Follow the patient back to the room when he gets out of his room	121	68	40
13	Regulate the possibility of smoking	158	34	39
14	Providing structure for the patient	17	52	158
15	Testing out that the patient is in the shared environment	55	146	28
16	Correction and boundary setting	33	115	83
17	Calming down and reassuring the patient	10	78	143
18	The patient is only in seclusion for a few hours a day	175	37	14
19	The patient is taken into or enters the room himself to be in seclusion when necessary	94	99	37
20	There is a gradual cessation of seclusion	73	102	52
	**Endings of seclusion**	**No reason**	**Additional reason**	**Main reason**
1	The patient gets along with others in the shared environment when this is tested	70	74	69
2	There is a reduction in the patient's symptoms	43	35	135
3	The patient's behavior has changed positively	35	51	128
4	The patient cooperates and keeps agreements	47	93	74
5	Patients or relatives have complained about the seclusion and got approval	207	0	3

**Table 2:** Decisions on checklist items based on rating of episodes and analyses of psychometric properties.

	**Reasons for seclusion (reduced to 6 items)**	**Decisions**	**Comments**
1	The patient's behavior affects other patients in a negative way	Remove	Unclear. Covered by items 2, 8, 9
2	The patient shows uncritical behavior	Keep	
3	The patient is intoxicated, and this affects the behavior	Remove	Covered by a factor with items 4–7
4	The patient is violent toward the staff	Merge	Merge with 5
5	The patient is threatening the staff	Merge	Merge with 4
6	The patient is violent toward other patients	Merge	Merge with 7
7	The patient is threatening other patients	Merge	Merge with 6
8	The patient's behavior is chaotic	Keep	
9	The patient has significantly increased activity	Keep	
10	Staff consider that there is a high risk of suicide	Keep	
	**Elements of seclusion (reduced to 10 items)**	**Decisions**	**Comments**
1	Activities with staff during seclusion	Merge	Merge in general item on activities
2	Activities with staff outside the ward	Merge	Merge in general item on activities
3	Activities alone during seclusion	Merge	Merge in general item on activities
4	Support conversations with the patient	Keep	
5	Reduction of stimuli or sensory impressions	Keep	Reformulated
6	Locking of personal belongings	Merge	Merge with 11
7	Regulation of access to TV, radio, or internet	Remove	Covered by reformulated 5
8	Regulation of contact with relatives	Merge	Merge in general item on contact
9	Regulation of contact with other patients	Merge	Merge in general item on contact
10	Regulation of access to mobile phone	Merge	Merge in general item on contact
11	Restrict access to objects that the patient can use to harm themselves or others	Merge	Merge with 6
12	Follow the patient back to the room when he gets out of his room	Keep	Merge 12, 15, 18, 19, 20
13	Regulate the possibility of smoking	Remove	More related to health as the reason
14	Providing structure for the patient	Keep	
15	Testing out that the patient is in the shared environment	Merge	Merge 12, 15, 18, 19, 20
16	Correction and boundary setting	Keep	
17	Calming down and reassuring the patient	Keep	
18	The patient is only in seclusion for a few hours a day	Merge	Merge 12, 15, 18, 19, 20
19	The patient is taken into or enters the room himself to be in seclusion when necessary	Merge	Merge 12, 15, 18, 19, 20
20	There is a gradual cessation of seclusion	Merge	Merge 12, 15, 18, 19, 20
	**Endings of seclusion (removed from the checklist)**	**Decisions**	**Comments**
1	The patient gets along with others in the shared environment when this is tested	Remove	Remove the whole section
2	There is a reduction in the patient's symptoms	Remove	Remove the whole section
3	The patient's behavior has changed positively	Remove	Remove the whole section
4	The patient cooperates and keeps agreements	Remove	Remove the whole section
5	Patients or relatives have complained about the seclusion and got approval	Remove	Remove the whole section

**Table 3:** Interrater reliability^*^ for 31 clinicians rating 20 seclusion episodes (written descriptions) with yes/no.

	**Reasons for seclusion**	**Exact agreement**	**Gwet's AC2**
1	The patient shows uncritical behavior	82 %	0.78
2	The patient shows chaotic behavior	72 %	0.53
3	The patient has significantly increased activity	70 %	0.46
4	The patient is threatening or violent toward staff	86 %	0.76
5	The patient is threatening or violent toward other patients	70 %	0.41
6	There is a high risk of suicide or severe self-harm	96 %	0.95
	**Elements of seclusion**		
1	Regulating the patient contacting others	89 %	0.87
2	Restricting access to objects	83 %	0.76
3	Regulating impressions	86 %	0.83
4	Calming down and reassuring the patient	83 %	0.79
5	Correcting or setting boundaries	73 %	0.61
6	Providing structure for the patient	77 %	0.67
7	Activities with staff	76 %	0.57
8	Supportive conversations with the patient	66 %	0.39
9	Following the patient back to the seclusion area	69 %	0.40
10	Gradually increasing the time in the shared environment	77 %	0.54

* *Grading of interrater reliability: 0.21–0.40 fair, 0.41–60 moderate, 0.61–0.80 substantial, 0.81–1.00 excellent*.

### Stage 5. Testing and Achieving Adequate Interrater Reliability of the Checklist

Statistical analyses of interrater reliability for 69 pairs of clinical staff in 22 wards rating the same seclusion episode using the revised checklist are reported in Supplementary Table 3 with comments. We found an acceptable level of agreement for most seclusion reasons using the dichotomous response scale (yes/no) but not for the seclusion elements rated using the graded response scale. Based on this and on comments from the participants in the project indicating that it was difficult to use the graded scale for several of the seclusion elements, we decided to revise the graded response scale for seclusion elements to a dichotomous scale (yes/no) and to perform an additional test of interrater reliability. The items were kept unchanged, and only the response scale for seclusion elements was changed.

Testing the interrater reliability for the revised checklist with dichotomous response scales also for seclusion elements was done with clinical staff rating written descriptions of 20 seclusion episodes from the original material collected in stage 1. The 20 descriptions were selected because they were detailed, covered all phases of seclusion episodes, and together covered different variations of seclusion episodes. Each description was shortened to a maximum of two pages by removing parts that were not necessary for scoring the checklist. A pilot test by two clinicians independently rating the 20 abbreviated descriptions indicated that it would be possible to obtain an acceptable agreement. The reliability testing was conducted in 2018 by 31 clinicians (5 doctors/psychologists and 26 from the milieu therapy staff). Interrater reliability of the ratings was analyzed using Gwet's AC for testing interrater reliability among multiple raters using a dichotomous response scale (43). The results are shown in Table 3. Gwet's AC showed moderate interrater reliability (0.41–0.60) for three reasons and substantial (0.61–0.80) or excellent (0.81–1.00) reliability for three reasons. The interrater reliability for seclusion elements was fair for two elements, moderate for two, and substantial or excellent for six. We concluded that the interrater reliability was acceptable for the checklist with dichotomous response scales.

### The Final Checklist

We considered the Clinical Seclusion Checklist to have acceptable content validity for seclusion in Norway and acceptable interrater reliability. It is brief and easy to complete, and it may be used in clinical work and research. The checklist is available as Supplementary Material (Data Sheet 2).

The final checklist includes four additional questions on contextual issues: formal decisions or voluntary agreement about seclusion, physical environment for the seclusion, whether staff are present, and the time point in the seclusion episode. The question on location of seclusion to the patient room or a seclusion area had been a part of the first Delphi round (Supplementary Table 1C), while the three other questions were added after the checklist had been tested.

To support a reliable understanding and rating of the items, we developed guidelines for rating the checklist with a brief explanation of each item. This was done based on the complete information from the different stages of the development and testing, supported by the clinical experience of project group members and feedback from participants in the project. The guidelines are available, together with the checklist, in the Supplementary Material (Data Sheet 2).

## Discussion

Summarizing the results, the Clinical Seclusion Checklist was developed and tested in a process of five stages. The thematic analyses of the large and detailed body of material identified potential seclusion reasons, elements and endings. The two-round Delphi process resulted in consensus regarding 10 reasons, 20 elements and five endings of seclusion considered to have good content validity from the first two stages. The first draft of the checklist with these items was tested rating a large number of seclusion episodes, and based on psychometric analyses of the results, the checklist was revised and shortened to six reasons and 10 elements of seclusion. Testing these items with dichotomous response scales resulted in the final checklist with acceptable interrater reliability.

### The Content of the Checklist

The number of seclusion reasons in the first part of the checklist was substantially reduced from the first list generated in stage 1. A large proportion of the seclusion reasons in the first Delphi round was related to securing the staff's work in the wards. However, the first Delphi round did not support that these were reasons for seclusion. The first Delphi round also showed clearly that no diagnosis in itself would suffice as a reason; rather, the patient's behavior would be the basis for seclusion. Thus, if a patient with schizophrenia was in seclusion, it would be due to his or her behavior and not to the diagnosis itself.

There may be one or more reasons for implementing seclusion, e.g. the patient may show chaotic behavior while also acting in a threatening manner. The first three reasons on the checklist are in regard to other disturbing behaviors rather than to a risk of harming oneself or others. In a nationwide 15-year study in Finland, agitation and disorientation were found to be the most frequent reasons for the use of seclusion and restraint, and this also supports the finding that both risk of harm and other disruptive behaviors may lead to the use of seclusion (44). Reasons 1–3 may be more associated with providing treatment, while reasons 4–6 are associated with the need to ensure safety and protect the patient from harming himself/herself or others.

The second part of the checklist comprises the elements of seclusion. These elements include both restrictions and support, representing aspects of containment as well as aspects of therapeutic intervention. Several of the items may contribute to both of these aims.

The checklist contains items on seclusion elements provided by the multidisciplinary milieu therapy staff but not items on specific treatments provided by psychiatrists or psychologists as part of psychiatric intensive care (1, 3). Psychotropic medication is a coercive measure when given as involuntary medication. However, we do not consider this as an element of seclusion, as psychotropic medication is also given as a coercive measure to involuntary admitted patients who are not secluded (45–48). For a complete picture of the total psychiatric intensive care, the checklist needs to be combined with other measurement tools.

The checklist does not measure the nature or quality of the interaction and communication between staff and patients; nor does it measure staff attitudes in their interactions with patients. It may be useful to combine the checklist with other measurement tools, such as the questionnaire on the Safewards interventions with focus on the interaction between staff and patients (10, 34) and/or the Staff Attitudes to Coercion Scale with focus on staff attitudes (49), even if these questionnaires in their present form are not rated regarding the interaction with a specific patient.

The four additional items on the context of seclusion represent dimensions that have been included in some definitions of seclusion. Including these four aspects in the checklist makes it possible to identify similarities and differences when comparing the use of seclusion across different sites or psychiatric intensive care models. Other questions may also be added, depending on what topics a project or study aims to cover. The additional items on context are considered to make the checklist feasible and useful in other settings and countries as well.

The checklist is not a definition of seclusion. However, it contains elements that were recognized as components of seclusion by clinical experts in Norway through the extensive development of the checklist with a large number of detailed descriptions of seclusion episodes, a nationwide consensus on elements, and a testing of the use of the elements in clinical practice.

### The Methods for the Development of the Checklist

As described above under methods and results, we consider the first three stages to ensure that the checklist had adequate content validity by adequate sampling and a set of items that reflects the clinical variation of seclusion in Norway (40). The input in all stages was from multiprofessional groups with both psychiatrists and clinical psychologists who make decisions on seclusion and mental health nurses and others who implement seclusion in practice.

Revising the first checklist, we followed well-established procedures with psychometric analyses of results from the clinical testing of the first version of the checklist (40, 41). Factor analyses demonstrated clear factor structures for both seclusion reasons and seclusion elements, and analyses of internal consistency and correlations between items in each factor gave further support for groups of items that could be replaced by a new item. Examination of the item contents was helpful to find more precise and shorter formulations for several items while still keeping the revised and shorter checklist true to the content validity achieved in the first stages of the checklist's development.

We achieved acceptable interrater reliability in the last stage of the checklist's development. However, our aim to achieve acceptable interrater reliability for a graded response scale for the seclusion elements was unsuccessful. It might have been possible if we had tried to create different response scales tailored to each element. To keep the checklist short and easy to complete, we wanted to have the same generic response scale for all 10 items. However, if a graded response scale should be considered more useful, it may be possible to redesign and test a graded response scale again for another version of the checklist.

As we have not found any other tool for measuring reasons for or elements of seclusion for the individual patient, we have not been able to conduct any test of constructive validity by comparing the checklist to a similar measurement tool. The criterion validity and construct validity of the checklist may be tested in future research.

Ideas for further development of the checklist may be to develop versions as questionnaires for patients and for family/relatives, to validate the checklist and other measurement tools by comparison with each other, to revise (add, remove, change) elements in the checklist based on new knowledge or studies, and to revise the guidelines for the checklist.

### Potential Use of the Checklist in Clinical Work and Research

Coercive measures shall be implemented only when necessary, under certain circumstances. It is essential to examine and measure how seclusion as a clinical and legal intervention is carried out. The checklist may contribute to awareness and reflection on the need for seclusion or necessary elements of seclusion in clinical practice and in quality assurance. Application of the checklist during a seclusion episode or at the end of an episode can contribute to the assessment of whether the reasons for seclusion are still present and if the elements of seclusion are still necessary. Some find any checklist as an unwanted workload in routine practice, while others may find a short checklist clinically useful and that it may be a time reducing evaluation of the daily clinical practice. As a part of a department's R&D practice, the checklist is short enough to be used for a simple measurement of the seclusion practice in specific time periods or projects which are beneficial for the clinical work.

Reporting on seclusion episodes based on the checklist may provide more valid and reliable reported data and more details on reasons for seclusion and how it is implemented. The checklist may also contribute to comparisons across sites and periods. Reported data that is more reliable will result in more-reliable national statistics as bases for mental health policy-making, recommendations, guidelines, regulations, and legislation on seclusion. If seclusion elements also are used based on voluntary agreements with patients, the checklist may help identify certain similarities and differences between the voluntary approach and seclusion as a coercive measure.

The checklist may be used in a range of research studies. More reliable measurements in descriptive studies may contribute to better data on the use of seclusion, including elements of seclusion. Cross-sectional studies comparing the content of seclusion across sites, models of care or countries may generate new knowledge about similarities and differences. Longitudinal studies may test changes over time in seclusion practices. Clinical trials may study the relationship between the content of seclusion and clinical outcome and the patient experience of seclusion. The review on effects of seclusion and restraints found that the only three existing randomized controlled trials (RCTs) showed that it is difficult to conduct such studies on coercion without a high risk of bias, and that this raises the question of whether RCT is an adequate design when studying the effects of seclusion (21). The authors of the review suggested that well-conducted prospective cohort studies of coercion could be more feasible and useful and have a greater clinical impact.

Overall, the variation in seclusion models within and across countries suggests the need for a framework and a uniform registration system for systematic comparison and monitoring of seclusion and its different dimensions (16). The Clinical Seclusion Checklist is a first step toward achieving more reliable measurements of seclusion, and it may be one building block in a uniform registration system that may be widely used. The value of the checklist will increase with an increasing amount of comparative results from different settings and models of seclusion, and from studies with various research questions. A combination of the checklist and other measurement tools would also contribute valuable information (e.g. the association between seclusion elements and use of antipsychotic medication), and further development of the checklist may also increase its usefulness. In particular, further studies that assess the predictive validity of the scale can provide refinement of the instrument and expand its potential utility.

### Strengths and Limitations

Strengths of the project include the large and representative body of material used to identify seclusion reasons and elements, the representative sample of participants in the Delphi process, and the extensive testing of the clinical relevance of the first draft of the checklist. The checklist was developed using a transparent process, making it possible to replicate the study and compare results. The project and the checklist also have several limitations. The checklist has dichotomous and not graded response scales. It measures only reasons for seclusion and elements of seclusion, and not how the elements are implemented, the attitudes of the staff, or the interaction between patients and staff.

## Conclusions and Implications

The Clinical Seclusion Checklist (CSC) is a brief and feasible tool with acceptable content validity and interrater reliability for measuring seclusion reasons and elements. The brevity of the checklist makes it feasible to be combined with other clinical measurement tools. It may be used to increase awareness of decisions and practices of seclusion, to compare seclusion practice across sites, for quality improvement of seclusion, for more valid and reliable reporting of seclusion episodes, and for research on the effects of seclusion and patient experiences of seclusion.

## Data Availability Statement

The data supporting the conclusions of this article will be made available by the first author upon reasonable request.

## Author Contributions

TH was the initiator of the project. TR and TH designed the methods and led the development of the checklist. TH led the sessions in the Network for Acute Mental Health Services where the project and results were discussed in each of the five stages. EH was a member of the project group throughout the project, participated in analyzing the descriptions of seclusion episodes, organized the interrater reliability testing, and wrote the instructions for the checklist. TR was the project manager, did the data analyses, and drafted the manuscript. HP advised on the Delphi process. All authors took part in writing the manuscript, and all authors have approved the final version.

## Funding

The project has been funded as a part of the Network for Acute Mental Health Services, including an additional support from the Regional Health Authority South-East, and the work of those who contributed to the project.

## Conflict of Interest

The authors declare that the research was conducted in the absence of any commercial or financial relationships that could be construed as a potential conflict of interest.

## Publisher's Note

All claims expressed in this article are solely those of the authors and do not necessarily represent those of their affiliated organizations, or those of the publisher, the editors and the reviewers. Any product that may be evaluated in this article, or claim that may be made by its manufacturer, is not guaranteed or endorsed by the publisher.
