# Supplementary material for: Measuring Seclusion in Psychiatric Intensive Care: Development and Measurement Properties of the Clinical Seclusion Checklist
Source: Front Psychiatry. 2021 Dec 23;12:768500. doi: 10.3389/fpsyt.2021.768500 (PMC8733687; doi:10.3389/fpsyt.2021.768500)
Supplement: Supplementary file 1 [file Table_1.docx]

**Supplementary table 1A. Assessment of reasons for seclusion. First Delphi round (47 participating wards)**

|  | **Reasons for seclusion** | **Rating *** | | | **Conclusion** |
| --- | --- | --- | --- | --- | --- |
|  | **Patient behavior** | **1-3** | **4-6** | **7-9** |  |
| 1 | Patient behavior affects fellow patients | 4 | 18 | 25 | Reformulated |
| 2 | The patient shows uncritical behavior | 1 | 11 | 35 | Retained |
| 3 | The patient is intoxicated | 6 | 11 | 30 | Reformulated |
| 4 | The patient does things the staff fears he will regret | 16 | 18 | 13 | Removed |
| 5 | It is difficult for the patient to comply with the ward rules | 29 | 15 | 3 | Removed |
| 6 | Staff consider that there is a high risk of suicide or self-harm | 25 | 11 | 11 | Reformulated |
| 7 | The patient is violent, aggressive, or threatening towards staff | 2 | 3 | 41 | Divided into two |
| 8 | The patient is violent, aggressive, or threatening towards other patients | 1 | 1 | 45 | Divided into two |
| 9 | The patient's behavior seems incomprehensible, irrational or chaotic | 3 | 22 | 21 | Reformulated |
| 10 | The patient has symptoms of psychosis | 31 | 11 | 5 | Removed |
| 11 | The patient has symptoms of mania | 9 | 11 | 27 | Removed |
|  | **Need for a better framework in observation or treatment** |  |  |  |  |
| 1 | Seclusion is a consequence of unacceptable behavior | 24 | 16 | 7 | Removed |
| 2 | One will start or secure medication | 43 | 4 | 0 | Removed |
| 3 | Prevent the patient from substance abuse in the ward | 40 | 7 | 0 | Removed |
| 4 | There is a need to observe the patient more closely | 43 | 3 | 1 | Removed |
| 5 | It is difficult to achieve a dialogue with the patient | 47 | 0 | 0 | Removed |
| 6 | Need seclusion to prevent the patient absconding | 41 | 3 | 3 | Removed |
| 7 | The patient needs more staff available outside seclusion | 41 | 5 | 1 | Removed |
| 8 | Prevention of unfortunate incidents that staff fear may occur | 18 | 21 | 7 | Removed |
| 9 | There is a desire to accelerate improvement for the patient | 14 | 11 | 22 | Removed |
|  | **Other possible reasons** |  |  |  |  |
| 1 | The patient wants seclusion | 25 | 11 | 11 | Retained |
| 2 | Seclusion has worked well on the patient in the past | 16 | 16 | 15 | Reformulated |
| 3 | Observed positive effect of seclusion-like measures | 23 | 7 | 15 | Removed |

*) Rating on a scale from 1 (no reason for seclusion) to 9 (certainly a reason for seclusion).

**Supplementary table 1B. Assessment of seclusion elements. First Delphi round (47 participating wards)**

|  | **Elements of seclusion** | **Rating *** | | | **Conclusion** |
| --- | --- | --- | --- | --- | --- |
|  | **Activities** | **1-3** | **4-6** | **7-9** |  |
| 1 | Activities with staff inside the seclusion area | 10 | 7 | 30 | Reformulated |
| 2 | Activities with staff outside the ward | 5 | 9 | 33 | Retained |
| 3 | Activities alone in seclusion | 9 | 10 | 27 | Retained |
| 4 | Supportive conversations with the patient | 10 | 7 | 30 | Retained |
|  | **Restrictions** |  |  |  |  |
| 1 | Reduction of stimuli or sensory impressions | 0 | 3 | 44 | Retained |
| 2 | Locking of personal belongings | 7 | 12 | 28 | Retained |
| 3 | Regulation of access to TV, radio, or internet | 3 | 6 | 38 | Retained |
| 4 | Regulation of contact with relatives | 12 | 10 | 24 | Retained |
| 5 | Regulation of contact with other patients | 2 | 3 | 41 | Retained |
| 6 | Regulation of access to mobile phone | 9 | 6 | 32 | Retained |
| 7 | Limit access to objects that the patient may use to harm themselves or others | 3 | 7 | 37 | Retained |
| 8 | Follow the patient back to the room when he gets out of his room | 5 | 4 | 38 | Retained |
| 9 | Regulate the possibility of smoking | 20 | 10 | 16 | Retained |
| 10 | Use of coercive measures | 22 | 9 | 15 | Removed |
|  | **Structure / treatment** |  |  |  |  |
| 1 | Provide structure for the patient | 15 | 7 | 25 | Retained |
| 2 | Testing the patient in a shared milieu with other patients | 8 | 12 | 27 | Retained |
| 3 | Assist the patient with boundary setting or correction | 11 | 11 | 25 | Reformulated |
| 4 | Assist the patient with daily chores | 22 | 8 | 17 | Removed |
| 5 | Calm down and reassure the patient | 13 | 8 | 26 | Retained |
| 6 | Observation of the patient's symptoms and behavior as part of an assessment or setting diagnosis | 30 | 4 | 12 | Removed |
| 7 | The patient is in seclusion only for a few hours a day | 13 | 9 | 25 | Retained |
| 8 | The patient is taken into or enters himself into the seclusion area if necessary | 15 | 10 | 22 | Retained |

*) Rating on a scale from 1 (not an element of seclusion) to 9 (certainly an element of seclusion).

**Supplementary table 1C. Assessment of seclusion contexts. First Delphi round (47 participating wards)**

|  | **Contexts of seclusion** | **Rating *** | | | **Conclusion** |
| --- | --- | --- | --- | --- | --- |
|  |  | **1-3** | **4-6** | **7-9** |  |
| 1a | The frameworks/context are individually adapted to each patient | 1 | 2 | 44 | Removed |
| 1b | Patients are involved in determining the framework/context for seclusion | 11 | 22 | 14 | Removed |
| 2a | The patient is in seclusion in a normal patient room | 10 | 9 | 28 | Removed |
| 2b | The patient is in seclusion in an seclusion area | 6 | 3 | 37 | Removed |

*) Rating on a scale from 1 (not an element of seclusion) to 9 (certainly an element of seclusion).

**Supplementary table 1D. Assessment of seclusion endings. First Delphi round (47 participating wards)**

|  | **Endings of seclusion** | **Rating *** | | | **Conclusion** |
| --- | --- | --- | --- | --- | --- |
|  |  | **1-3** | **4-6** | **7-9** |  |
| 1 | The patient gets along with others in the shared environment when this is tried out | 0 | 5 | 42 | Retained |
| 2 | Patient has improved, or symptom reduction | 0 | 4 | 43 | Retained |
| 3 | The patient cooperates and keeps agreements | 0 | 14 | 33 | Retained |
| 4 | Practical reasons | 31 | 6 | 10 | Removed |
| 5 | Decisions expire and one chooses not to renew it | 23 | 10 | 14 | Removed |
| 6 | The patient likes seclusion | 25 | 14 | 8 | Removed |

*) Rating on a scale from 1 (not an element of seclusion) to 9 (certainly an element of seclusion).

**Supplementary table 1E. Assessment of what seclusion elements which may be used in milieu therapy also outside seclusion. First Delphi round (47 participating wards)**

|  | **Elements of seclusion** | **Rating *** | | | **Conclusion** |
| --- | --- | --- | --- | --- | --- |
|  | **Activities** | **1-3** | **4-6** | **7-9** |  |
| 1 | Activities with staff inside the seclusion area | 0 | 0 | 47 | Yes |
| 2 | Activities with staff outside the ward | 0 | 0 | 47 | Yes |
| 3 | Activities alone in seclusion | 0 | 1 | 44 | Yes |
| 4 | Supportive conversations with the patient | 0 | 0 | 47 | Yes |
|  | **Restrictions** |  |  |  |  |
| 1 | Reduction of stimuli or sensory impressions | 5 | 19 | 23 | Partially |
| 2 | Locking of personal belongings | 5 | 8 | 34 | Partially |
| 3 | Regulation of access to TV, radio, or internet | 10 | 13 | 24 | Partially |
| 4 | Regulation of contact with relatives | 11 | 6 | 29 | Partially |
| 5 | Regulation of contact with other patients | 16 | 13 | 17 | Partially |
| 6 | Regulation of access to mobile phone | 8 | 6 | 33 | Partially |
| 7 | Limit access to objects that the patient may use to harm themselves or others | 8 | 11 | 28 | Partially |
| 8 | Follow the patient back to the room when he gets out of it | 22 | 7 | 18 | Partially |
| 9 | Regulate the possibility of smoking | 7 | 8 | 31 | Partially |
| 10 | Use of coercive measures | 6 | 3 | 38 | Yes |
|  | **Structure / treatment** |  |  |  |  |
| 1 | Provide structure for the patient | 0 | 1 | 46 | Yes |
| 2 | Testing the patient in a shared milieu with other patients | 13 | 9 | 25 | Partially |
| 3 | Assist the patient with boundary setting or correction | 0 | 3 | 44 | Yes |
| 4 | Assist the patient with daily chores | 0 | 3 | 44 | Yes |
| 5 | Calm down and reassure the patient | 0 | 0 | 47 | Yes |
| 6 | Observation of the patient's symptoms and behavior as part of an assessment or setting diagnosis | 1 | 2 | 43 | Yes |
| 7 | The patient is in seclusion only for a few hours a day | 7 | 7 | 33 | Partially |
| 8 | The patient is taken into or enters himself into the seclusion area if necessary | 5 | 8 | 34 | Partially |

*) Rating on a scale from 1 (may not be used outside seclusion) to 9 (may certainly be used also outside seclusion).
