# Supplementary material for: Measuring Seclusion in Psychiatric Intensive Care: Development and Measurement Properties of the Clinical Seclusion Checklist
Source: Front Psychiatry. 2021 Dec 23;12:768500. doi: 10.3389/fpsyt.2021.768500 (PMC8733687; doi:10.3389/fpsyt.2021.768500)
Supplement: Supplementary file 2 [file Table_2.docx]

**Supplementary table 2A. Assessment of reasons for seclusion. Second Delphi round (41 participating wards)**

|  | **Reasons for seclusion** | **Rating *** | | | **Conclusion** |
| --- | --- | --- | --- | --- | --- |
|  |  | **1-3** | **4-6** | **7-9** |  |
| 1 | The patient's behavior affects other patients in a negative way | 1 | 6 | 34 | Retained |
| 2 | The patient needs to be protected from other patients (new) | 28 | 8 | 4 | Removed |
| 3 | The patient shows uncritical behavior | 0 | 2 | 39 | Retained |
| 4 | The patient is intoxicated, and this affects the behavior | 1 | 3 | 36 | Retained |
| 5 | Staff consider that there is a high risk of suicide | 23 | 8 | 10 | Retained |
| 6 | The staff considers that there is a high risk of self-harm | 30 | 9 | 2 | Removed |
| 7 | The patient is violent towards the staff | 2 | 1 | 38 | Retained |
| 8 | The patient is threatening the staff | 3 | 3 | 35 | Retained |
| 9 | The patient is violent towards other patients | 1 | 0 | 40 | Retained |
| 10 | The patient is threatening other patients | 1 | 0 | 40 | Retained |
| 11 | The patient's behavior is chaotic | 0 | 12 | 29 | Retained |
| 12 | The patient is confused (new) | 9 | 19 | 13 | Covered by 12 |

*) Rating on a scale from 1 (no reason for seclusion) to 9 (certainly a reason for seclusion).

**Supplementary table 2B. Assessment of seclusion elements. Second Delphi round (41 participating wards)**

|  | **Elements of seclusion** | **Rating *** | | | **Conclusion** |
| --- | --- | --- | --- | --- | --- |
|  | **Activities** | **1-3** | **4-6** | **7-9** |  |
| 1 | Activities with staff in seclusion | 3 | 3 | 35 | Retained |
| 2 | Activities with staff outside the ward | 4 | 4 | 33 | Retained |
| 3 | Activities alone in seclusion | 3 | 5 | 33 | Retained |
| 4 | Supportive conversations with the patient | 5 | 4 | 32 | Retained |
|  | **Restrictions** |  |  |  |  |
| 1 | Reduction of stimuli or sensory impressions | 0 | 1 | 40 | Retained |
| 2 | Locking of personal belongings | 3 | 4 | 34 | Retained |
| 3 | Regulation of access to TV, radio, or internet | 0 | 2 | 39 | Retained |
| 4 | Regulation of contact with relatives | 3 | 6 | 30 | Retained |
| 5 | Regulation of contact with other patients | 0 | 0 | 31 | Retained |
| 6 | Regulation of access to mobile phone | 2 | 2 | 37 | Retained |
| 7 | Limit access to objects that the patient may use to harm themselves or others | 3 | 0 | 38 | Retained |
| 8 | Follow the patient back to the room when he gets out of it | 2 | 2 | 37 | Retained |
| 9 | Regulate the possibility of smoking | 13 | 8 | 20 | Retained |
|  | **Structure / treatment** |  |  |  |  |
| 1 | Provide structure for the patient | 3 | 4 | 34 | Retained |
| 2 | Testing the patient in a shared environment with other patients | 6 | 4 | 31 | Retained |
| 3 | Boundary setting or correction | 1 | 9 | 31 | Retained |
| 4 | Calm down and reassure the patient | 4 | 3 | 34 | Retained |
| 5 | The patient is in seclusion only for a few hours a day | 9 | 5 | 26 | Retained |
| 6 | The patient is taken into his room or enters the room himself for seclusion if necessary | 7 | 4 | 39 | Retained |
| 7 | There is a gradual cessation of seclusion | 0 | 0 | 41 | Retained |

*) Rating on a scale from 1 (not an element of seclusion) to 9 (certainly an element of seclusion).

**Supplementary table 2C. Assessments of seclusion endings. Second Delphi round (41 participating wards)**

|  | **Endings of seclusion** | **Rating *** | | | **Conclusion** |
| --- | --- | --- | --- | --- | --- |
|  |  | **1-3** | **4-6** | **7-9** |  |
| 1 | The patient gets along with others in the shared environment when this is tried out | 0 | 1 | 40 | Retained |
| 2 | The patient has improved, or reduced symptoms | 0 | 0 | 41 | Retained |
| 3 | The patient's behavior has changed positively (new) | **0** | **2** | **39** | Retained |
| 4 | The patient cooperates and keeps agreements | **0** | **4** | **37** | Retained |
| 5 | Patients or relatives have complained about the seclusion and got approval (new) | **0** | **7** | **34** | Retained |
| 6 | Seclusion does not work for this patient (new) | 4 | 15 | 21 | Removed |
| 7 | The patient loses function as a result of seclusion | 4 | 12 | 22 | Removed |

*) Rating on a scale from 1 (not an ending of seclusion) to 9 (certainly an ending of seclusion).
