# Supplementary material for: Measuring Seclusion in Psychiatric Intensive Care: Development and Measurement Properties of the Clinical Seclusion Checklist
Source: Front Psychiatry. 2021 Dec 23;12:768500. doi: 10.3389/fpsyt.2021.768500 (PMC8733687; doi:10.3389/fpsyt.2021.768500)
Supplement: Supplementary file 3 [file Table_3.docx]

**Supplementary table 3. Interrater reliability* for pairs of independent ratings of 69 seclusion episodes**

|  |  | **ICC** | **Kappa** | **Weighted kappa** |
| --- | --- | --- | --- | --- |
|  | **Reasons for seclusion (dichotomous response scale)** |  |  |  |
| 1 | The patient shows uncritical behavior | 0.34 | 0.55 | - |
| 2 | The patient shows chaotic behavior | 0.28 | 0.42 | - |
| 3 | The patient has significantly increased activity | 0.30 | 0.49 | - |
| 4 | The patient is threatening or violent towards staff | 0.37 | 0.63 | - |
| 5 | The patient is threatening or violent towards other patients | 0.22 | 0.17 | - |
| 6 | There is high risk of suicide or serious self-harm | - | 1.00 | - |
|  | **Elements of seclusion (graded response scale)** |  |  |  |
| 1 | Regulating the patient contacting others | 0.26 | 0.21 | 0.28 |
| 2 | Restricting access to objects | 0.52 | 0.23 | 0.40 |
| 3 | Regulating impressions | 0.27 | 0.27 | 0.32 |
| 4 | Calming down and reassuring the patient | 0.19 | 0.02 | 0.15 |
| 5 | Correcting or setting boundaries | 0.47 | 0.24 | 0.38 |
| 6 | Providing structure for the patient | 0.21 | 0.08 | 0.15 |
| 7 | Activities with staff | 0.36 | 0.14 | 0.30 |
| 8 | Supportive conversations with the patient | 0.21 | 0.11 | 0.15 |
| 9 | Following the patient back to the seclusion area | 0.21 | 0.37 | 0.40 |
| 10 | Gradually increasing the time in the shared environment | 0.43 | 0.29 | 0.39 |

*) Grading of kappa: 0.21-0.40 usable, 0.41-60 moderate, 0.61-0.80 substantial, 0.81-1.00 excellent.

ICC was run in the SAS program. Kappa was run in SPSS for elements, and in STATA for reasons and weighed kappa.

Cohen’s weighted kappa was calculated with linear weights for the distance between scores. For seclusion reasons with dichotomous responses (yes/no) only two of the methods have been used, since Cohen's weighted kappa can only be used for graded response scale, like for seclusion elements.

Kappa was unacceptable for one reason, moderate for three reasons, substantial for one reason and excellent for one reason. Weighted kappa was unacceptable for three elements, usable for seven elements, and not moderate, substantial or excellent for any elements.
