# Supplementary material for: Measuring Seclusion in Psychiatric Intensive Care: Development and Measurement Properties of the Clinical Seclusion Checklist
Source: Front Psychiatry. 2021 Dec 23;12:768500. doi: 10.3389/fpsyt.2021.768500 (PMC8733687; doi:10.3389/fpsyt.2021.768500)
Supplement: Supplementary file 4 [file Data_Sheet_1.pdf]

Form and guidelines for description of a seclusion episode

Systematic description of an episode of seclusion or another situation where a patient is separated from other patients, with or without a decision regarding seclusion

Institution		Formal decision has been done regarding seclusion	☐ Yes		☐ No
Department		Duration of seclusion	Days	Hours	Minutes
Ward		Contact person with phone number			

Contact information for the project management

Project manager (Name of project manager), e-mail address
Project coordinator (Name of coordinator), phone number, email address

Submission of completed forms is done by secure uploading on the website.

Guidelines for a systematic description of an episode of seclusion

1. The quality of the descriptions is essential for the success of the project. Please write as specific, detailed, and complete as possible in each section.
2. Each ward describes three (or hopefully more) episodes of seclusion during the period 10.09.12 – 09.12.12. Wards with less than three, describes the existing seclusions.
3. Each description is done together with the therapists and milieu staff who have taken part in decision making and implementation of the seclusion. The description should preferably be done by more than one person. Everyone should read the project description to understand the project.
4. No dates or other information that may identify the patient should be registered.
5. The description is written in THIS registration form. Each section of the form may be shortened or extended as needed by deleting or adding lines. Completed form is submitted as a word-file.
6. Parts of the description may be retrieved from medical records and registered reports, but these have to be supplemented with more accurate and complementary information customised to the topic.
7. Describe concretely and in detail the measures undertaken for seclusion and the course of the episode of the seclusion. This applies to what patients and staff say and do, considerations, decisions, measures implemented, physical surroundings where the seclusion takes place, and any other relevant information.
8. Describe the situation and behaviour concretely, so that others can picture it. Don’t use abstract words like agitated, disruptive, aggressive, psychotic, and upset.
9. The form is partly structured but does not contain cues for everything that may be applicable. Include everything you think is relevant, even if it’s not requested in the form.
10. It is the local coordinator in the department who submits the completed form. The project management may contact you if they need further clarification of the description.
11. Local coordinator and those who complete the form may contact the project management with questions or in the need of clarifications.

**<u>Basis and reason</u> that a patient is been in seclusion or kept separated from other patients:** Give as detailed description of the situation and events as you consider necessary for others to identify a similar situation or event.
**<u>Goals for the seclusion</u>**: Give as detailed description of the goals for the seclusion as you consider necessary in order for others to use it in a similar way. If the goals change during the course, this should also be described.
**<u>Seclusion-measures</u>:** Describe where the patient is, which constraints are done, who is with the patient, and what the staff do together with the patient. Have in mind that others, based on this description, should be able to conduct similar seclusion and arrangements like you have done. If there have been various measures in different phases, these various measures and different phases should be described. In addition, a short description of the premises, physical surroundings or other context is wanted.

**<u>Ethical aspects</u>:** Describe ethical considerations, how they have influenced thoughts and actions, and how this has been perceived by the patient and the staff. Sometimes moral conception become more difficult because one experience dilemmas between contradictory values. Did such moral challenges or dilemmas occur during the seclusion? Describe how this has been handled in practise and what has been the decisive factors for the choices made. Describe also the degree of consensus between the staff and patient, as well as agreement or disagreement among the staff.
**<u>Termination of the seclusion</u>:** Describe the duration of the seclusion, reason for termination of the seclusion, and how the termination was implemented
**Describe <u>any additional information</u> not covered in the sections above**
