# Supplementary material for: Measuring Seclusion in Psychiatric Intensive Care: Development and Measurement Properties of the Clinical Seclusion Checklist
Source: Front Psychiatry. 2021 Dec 23;12:768500. doi: 10.3389/fpsyt.2021.768500 (PMC8733687; doi:10.3389/fpsyt.2021.768500)
Supplement: Supplementary file 5 [file Data_Sheet_2.pdf]

Clinical Seclusion Checklist for rating by staff in psychiatric intensive care

Patient ______ ______ ______

Rater ______ **Date** ______

Time period for rating: Last 24 hours ☐ Other: ______

**Reasons for starting or continuing seclusion** (mark yes or no)		**Yes**	**No**
R1	The patient shows uncritical behavior	☐	☐
R2	The patient shows chaotic behavior	☐	☐
R3	The patient has significantly increased activity	☐	☐
R4	The patient is threatening or violent towards staff	☐	☐
R5	The patient is threatening or violent towards other patients	☐	☐
R6	There is high risk of suicide or serious self-harm	☐	☐

**Seclusion element used** (mark yes or no)		**Yes**	**No**
E1	Regulating the patient contacting others	☐	☐
E2	Restricting access to objects	☐	☐
E3	Regulating impressions	☐	☐
E4	Calming down and reassuring the patient	☐	☐
E5	Correcting or setting boundaries	☐	☐
E6	Providing structure for the patient	☐	☐
E7	Activities with staff	☐	☐
E8	Supportive conversations with the patient	☐	☐
E9	Following the patient back to the seclusion area	☐	☐
E10	Gradually increasing the time in the shared environment	☐	☐

C1 Seclusion by formal decision or agreement ☐ Formal decision ☐ Voluntary agreement

C2 Place where patient is in seclusion ☐ Patient room ☐ Seclusion area

C3 Patient is together with staff in seclusion ☐ Yes ☐ No

C4 Point in seclusion episode ☐ Beginning ☐ Middle ☐ End

Clinical Seclusion Checklist version 1.0

Supplementary material to: Ruud, Haugom, Pincus and Hynnekleiv: Measuring Seclusion in Psychiatric Intensive Care: Development and Measurement Properties of the Clinical Seclusion Checklist (Frontiers in Psychiatry, 2021)

Guidelines to staff for completing the Seclusion Clinical Checklist for psychiatric intensive care

These guidelines should be read before using the Seclusion Clinical Checklist. The purpose of the guidelines is to describe more in detail the reasons and elements in the checklist. The text below is based on 149 descriptions of seclusion episodes collected for the development of the checklist.

Reasons why seclusion is started or continued

The decision on providing seclusion may have several reasons. Mark Yes for each reason for the decision.

R1. The patient shows uncritical behavior
The patient undresses, tells private things (e.g., personal announcements and stories about their delusions), speaking loudly in a shared environment or seeking contact, and the behavior negatively affects other patients or staff.

R2. The patient shows chaotic behavior
The patient's behavior seems incomprehensible, irrational, or disorganized (e.g., showering with clothes on, disassembling things, tampering with electrical items)

R3. The patient has significantly increased activity
The patient is restless, more active than usual, seeking contact, and asking many questions. He/she has difficulty relaxing and is walking a lot.

R4. The patient is threatening or violent towards staff
Threats, such as saying that staff or someone close to them should be harmed. Threatens to punch or hit staff. The degree of threats and the patient's ability to realize these are factors to be considered. Violent behavior is physical attacks, such as punches, kicks, headbanging).

R5. The patient is threatening or violent towards other patients
Behavior like of 4 above, but towards other patients.

R6. There is a high risk of suicide or severe self-harm
The risk of suicide or severe self-harm may be such that it is difficult to follow up in a regular ward milieu, and seclusion may be necessary to secure the patient.

Assessment of seclusion elements

Mark Yes for each of the elements used in the specific seclusion episode. (Some elements are natural elements in milieu therapy in psychiatric wards and may therefore also be practiced outside seclusion.)

E1. Regulating the patient contacting others
This can apply to relatives, patients, or staff. Regulation of connections to the outside world (receiving visits, using telephone, sending/receiving letters, use of the internet, etc.). Regulation of contact with others should be marked regardless of whether a formal decision has been made.

E2. Restrict access to objects
This may include conditions such as staff locking up personal belongings or removing objects from the room that the patient could use to harm themselves or others.

E3. Regulating impressions
Regulation of access to TV, radio, reading material, PC, or internet. Regulation of the number of staff who contact the patient, limiting how many and whom the patient must relate to. Having time alone, time for resting, and neutral rooms are further examples.

E4. Calming down and reassuring the patient
Providing safety by regulating staff presence or distance to give the patient "space" if the patient so desires. If possible, it is done by staff well known to the patient, and if necessary, with more staff present. This may include relaxation techniques/diversions of attention that the patient has expressed is a help to calm down. Supportive conversations may also contribute, but should be marked on element 8.

E5. Correcting and setting boundaries
Set a framework for what is allowed. This can be done by guiding, making agreements, and training in setting own boundaries. House rules are also an example of expected or acceptable behavior.

E6. Providing structure for the patient
Help the patient to structure everyday life. This can include waking up in the morning, planning the day and time, setting goals for the stay, and the like. A daily plan is an example, and it can contain the day's chores such as meals, conversations, times for medication, and activities.

E7. Activities with staff
This can occur inside the seclusion area and include games, music, card games, reading, or other activities. Or it can take place outside the seclusion area or the ward, such as walking, working out in the gym, ball games, etc.

E8. Supportive conversations with the patient
The supportive conversation is planned and structured. This is often a conversation about what the patient finds difficult in the here-and-now situation. The patient may have a lot on his mind, may express a need to relieve himself, and the staff makes themselves available.

E9. Follow the patient back to the seclusion area
This is carried out if the patient is unable to relate to the other people in a shared environment or seclusion area. It can be done voluntarily by cooperation, or if necessary, following the patient back to their room against their own will.

E10. Gradual increasing the time in the shared environment
Test the patient in the shared environment before the seclusion is ended. If the seclusion is ended without a gradual increase in advance, "No" is marked.
